# Prolonged Survival of Subcutaneous Allogeneic Islet Graft by Donor Chimerism without Immunosuppressive Treatment

**DOI:** 10.1155/2017/7057852

**Published:** 2017-06-21

**Authors:** Brend Ray-Sea Hsu, Shin-Huei Fu, Aline Yen Ling Wang

**Affiliations:** ^1^Division of Endocrinology and Metabolism, Department of Internal Medicine, Chang Gung Medical Center, Taoyuan, Taiwan; ^2^School of Traditional Chinese Medicine, College of Medicine, Chang Gung University, Taoyuan, Taiwan; ^3^Department and Graduate Institute of Microbiology and Immunology, National Defense Medical Center, Taipei, Taiwan; ^4^Center for Vascularized Composite Allotransplantation, Chang Gung Memorial Hospital, Taoyuan, Taiwan

## Abstract

The aim of this study was to investigate whether tolerance-induced protection of islets in the renal subcapsular space can also prevent subcutaneous allogeneic islets from being rejected. We used bone marrow stem cells from C57BL/6 (H2^b^) mice to construct donor chimerism in conditioned diabetic BALB/c (H2^d^) mice and investigated the effect of donor chimerism on engraftment and survival of subcutaneously transplanted allogeneic islets in streptozotocin-induced diabetic mice. We also studied the anti-inflammatory effect of mesenchymal stem cell on islet engraftment. Full but not low-grade or no donor chimerism was associated with successful engraftment of allogeneic islets and restoration of normoglycemia in the treated diabetic mice. The temporary hyperglycemia was 11 ± 1 versus 19 ± 5 days (*p* < 0.05) for the mice with full donor chimerism with transplanted islets in the renal subcapsular space versus the subcutaneous space, respectively. Cotransplantation of mesenchymal stem cell did not enhance alloislet engraftment. Full multilineage donor chimerism was associated with a higher transient expansion of CD11b^+^ and Gr-1^+^ myeloid progenitor cells and effector memory CD4 and CD8 T cells. In conclusion, full donor chimerism protected both renal subcapsular and subcutaneous allogeneic islets in this rodent transplantation model.

## 1. Introduction

Transplantation of islets into the subcutaneous space to treat diabetes has been studied for decades; however, it has yet to be applied clinically [1]. Compared to the renal subcapsular or intraportal space, the subcutaneous space is easier to approach for both islet implantation and biopsy; however, the characteristics of poor vascularization and lower oxygenation of the subcutaneous space mean that engraftment of islet cells is unfavorable [2]. Therefore, when the subcutaneous space is used for islet transplantation, many more islet cells are required to restore normoglycemia in diabetic recipients [3]. Many prevascularized devices have been developed to improve the engraftment of subcutaneous islet cells in the early stage of transplantation [4]. However, these devices have not been able to protect donor islet cells from the toxicity resulting from the long-term administration of immunosuppressants in diabetic recipients [5]. In order to avoid the need for the long-term administration of immunosuppressants, induction of tolerance to allogeneic islets to prevent immune rejection has been achieved by infusing bone marrow stem cells and/or mesenchymal stem cells [6]. Since mesenchymal stem cell (MSC) has been reported to modulate immunity and attenuate inflammatory reaction, we investigated the adjuvant effect of MSC on allogeneic islet engraftment [7, 8]. The majority of related studies have used either the renal subcapsular space or intraportal area to implant allogeneic islets [9]. In this study, we investigated whether immune tolerance-induced protection of islets in the renal subcapsular space can also prevent rejection of subcutaneous allogeneic islets. Therefore, we used bone marrow stem cells from C57BL/6 (H2^b^) mice to construct donor chimerism in conditioned diabetic BALB/c (H2^d^) mice and investigated the effect of donor chimerism on engraftment and survival of subcutaneously transplanted allogeneic islets in streptozotocin-induced diabetic mice.

## 2. Materials and Methods

All chemicals, including streptozotocin, histopaque-1077, and type XI collagenase, were obtained from Sigma Chemical Company (St. Louis, MO, USA). RPMI-1640 medium was purchased from GIBCO Invitrogen, Life Technologies, Inc. (Grand Island, NY, USA). Intravenous busulfan (Busulfex) was sourced from P.D.L. Bio Pharma, Inc. (Incline Village, NV, USA), and cyclophosphamide was supplied by Baxter (Deerfield, IL, USA). Anti-insulin antibodies were obtained from Abcam Inc. (Cambridge, MA, USA). Whole blood glucose was determined using a One Touch II portable glucometer (Lifescan Inc., Milpitas, CA, USA) using dry reagent technology based on the glucose oxidase method via tail snipping. Wharton's jelly-derived mesenchymal stem cells (WJ-MSC) were gifted by Dr. Ing-Kae Wang from the Industrial Technology Research Institute of Taiwan.

### 2.1. Animal Care and Induction of Diabetes

Inbred, 8 to 12 weeks old male BALB/c (H2^d^) and C57BL/6 (H2^b^) mice were supplied by local breeders. Three to five mice were housed in each cage and fed with a standard pelleted food and tap water ad libitum. The animal room had an automatic lighting cycle with 12 hours of light and 12 hours of darkness. To induce diabetes, the BALB/c (H2^d^) mice received a single intraperitoneal injection of streptozotocin 200 mg/kg body weight. The mice with a whole blood glucose level exceeding 360 mg/dL for more than 2 weeks were used as the diabetic recipients. The body weight and nonfasting whole blood glucose levels of the mice were determined twice a week between 8:00 AM and 10:00 AM. After islet transplantation, the cure of diabetes in the recipients was defined as a whole blood glucose levels maintained below 200 mg/dL for two consecutive tests and thereafter. The days of temporary hyperglycemia were calculated as the duration before diabetes was cured. All of the animals were treated humanely in accordance with the laboratory animal guidelines of Chang Gung Memorial Hospital.

### 2.2. Islet Isolation

Pancreatic islets were isolated from the C57BL/6 (H2^b^) mice by collagenase digestion, enriched on a histopaque density gradient, and finally hand-picked. Briefly, under anesthesia, the pancreases of the nonfasted healthy mice were distended with 2.5 mL RPMI-1640 medium containing 1.5 mg/mL collagenase and then excised and incubated in a 37°C water bath. The islets were purified using a density gradient and were hand-picked under a dissecting microscope. Isolated islets with diameters of 125–150 *μ*m were collected and counted into groups of 75 islets per group. The islets were then transplanted into the diabetic recipients on the day the islets were isolated. To minimize the influence of batch-to-batch variations in islet function on experimental observations, all islets isolated from 8–12 mice in a single day were separated equally and transplanted into the same number of mice in both the control and experimental groups on the day of isolation.

### 2.3. Renal Subcapsular Transplantation

The islets of C57BL/6 (H2^b^) mice were centrifuged in PE-50 tubing connected to a 200 *μ*L pipette tip. With the recipient BALB/c (H2^d^) mice under anesthesia, the left kidney was exposed through a lumbar incision. A capsulotomy was performed in the lower pole of the kidney, and the tip of the PE-50 tubing (Clay Adams, Parsippany, NJ, USA) was advanced beneath the capsule toward the upper pole, where the islet graft (300 islets for each recipient) was implanted using a Hamilton syringe. The capsule was left unsutured. The retroperitoneal cavity was closed using a two-layered suture.

### 2.4. Subcutaneous Islet Transplantation

The islets of C57BL/6 (H2^b^) mice were mixed with 30 *μ*L of Corning® Matrigel® (Corning Matrigel basement membrane matrix, Corning, NY, USA) and collected in a 1 mL syringe. Then, the islet mixture was injected subcutaneously in BALB/c (H2^d^) mice (450 islets in each site, 900 islets for each recipient) through a 25-gauge needle.

### 2.5. Preparation of Whole Bone Marrow Cells

With the donor C57BL/6 (H2^b^) mice under anesthesia, both femurs and tibiae were removed using a sterile procedure and bone marrow cells (BMCs) were flushed out of the bone into phosphate buffer solution (PBS). After being gently dispersed by pipetting cells in and out, whole bone marrow cells were washed twice in PBS and recovered by centrifugation at 1150 × *g* for 5 minutes at 4°C with a slow brake. Approximately 3 × 10^7^ BMCs in 0.2 mL PBS were intraorbitally injected into each recipient mouse immediately after preparation.

### 2.6. Standard Protocol of Nonmyeloablative Conditioning and Bone Marrow Cell Transplantation

The induction of donor chimerism in the BALB/c mice was modified from a previously described protocol [10]. Briefly, recipients were intraorbitally injected with busulfan, diluted in 0.9% normal saline to a final concentration of 1 mg/mL, at a dose of 10 mg/kg one day before islet transplantation. Intraorbital injections of 3 × 10^7^ C57BL/6 (H2^b^) bone marrow cells were given on the same day as islet transplantation (day 0). One day after islet transplantation, intraperitoneal injections of cyclophosphamide were given at a dose of 200 mg/kg of body weight (day 1). An additional intraorbital injections of 3 × 10^7^ allogeneic BMCs were given one day after the administration of cyclophosphamide (day 2). To reduce the adverse effects of cyclophosphamide on donor BMCs, the time between each BMC infusion and intraperitoneal injection of cyclophosphamide was strictly limited to 24 hours.

### 2.7. Experimental Design

**Figure 1:**
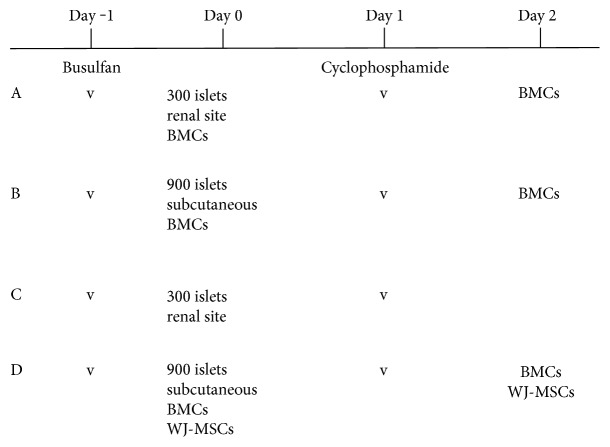
A diagrammatic timetable illustrating the experimental design and grouping. The day of islet transplantation is day 0. BMCs: bone marrow cells; WJ-MSC: Wharton's jelly-derived mesenchymal stem cells.

Mice in group A (*n* = 16) received a standard protocol of chimerism induction including conditioning with busulfan and cyclophosphamide and two infusions of bone marrow cells, followed by the implantation of 300 allogeneic islets into the subcapsular space of the left kidney. Mice in group B (*n* = 8) received the standard protocol of chimerism induction as in group A, except that each diabetic mouse received 900 islets implanted subcutaneously. Mice in group C (*n* = 6) were treated as those in group A but with no infusion of BMCs. Mice in group D (*n* = 6) were treated as per the mice in group B with two additional intravenous infusions of 2 × 10^5^ Wharton's jelly-derived mesenchymal stem cells (WJ-MSCs) given on the same day as BMC infusion. A diagrammatic timetable illustrating the experimental procedure and grouping is depicted in Figure 1.

### 2.8. Determination of Chimerism in the Peripheral Blood of Recipient Mice

Chimerism in the BALB/c mice was detected by staining for H2-D^b^-expressing (C57BL/6 donor) cells that were distinguishable from BALB/c cells expressing H2-D^d^. Other antibodies used for chimerism analysis including CD45, CD4, CD8, CD11b, CD11c, CD19, Ly6G/6C (Gr1), and Foxp3 were purchased from BD Biosciences (San Jose, CA, USA), eBioscience Inc., (San Diego, CA, USA), and BioLegend Inc., (San Diego, CA, USA). Peripheral blood was sampled to determine the degree of chimerism in the recipient mice. A volume of 0.15 mL of whole blood was collected in 0.5 mL of PBS containing 3 mg/mL of EDTA and 5 U/mL of heparin. After red blood cells had been lysed, approximately 2-3 × 10^5^ cells were incubated with different antibodies, and the cells were then washed and analyzed by flow cytometry using a FACSCalibur flow cytometer (Becton Dickinson, San Jose, CA, USA).

### 2.9. Determination of the Percentage of Effector Memory T Cells and Central Memory T Cells in the Peripheral Blood of Recipient Mice

We identified T cell subsets by flow cytometry analysis as naive T cells (TN, defined as CD44^lo^CD62L^+^), central memory T cells (TCM, defined as CD44^hi^CD62L^+^), and effector memory T cells (TEM, defined as CD44^hi^CD62L^−^). Antibodies were purchased from BD Biosciences (San Jose, CA), eBioscience (San Diego, CA), and BioLegend (San Diego, CA) and included anti-CD44 (IM7), anti-CD62L (MEL-14), anti-CD4 (RM4-5), and anti-CD8 (53.6.7). After primary staining, the cells were washed three times and then run on a FACSCalibur flow cytometer (Becton Dickinson, San Jose, CA, USA) and analyzed using FlowJo software (TreeStar, Ashland, OR).

### 2.10. Histological and Immunohistochemical Examination of the Retrieved Islet Grafts

Subcutaneous graft and graft-bearing left kidneys of the recipient mice were removed for histological examination at sacrifice. Immunohistochemical examinations of tissue sections were performed using anti-insulin antibodies. The secondary antibody was prepared from rabbits and conjugated with horseradish peroxidase, and 3,3′-diaminobenzidine was used as the chromogenic substrate.

### 2.11. Measurement of Pancreatic Insulin Content

Insulin content in the pancreas remnant was extracted using the acid-ethanol method. Briefly, the pancreases from nonfasted mice were removed randomly between 8:00 AM and 10:00 AM, homogenized in an acid-ethanol solution, and stored at 4°C overnight. After centrifugation at 2400 rpm for 30 minutes at 4°C, the supernatant was collected and stored at −20°C. The pancreas remnants were then homogenized again in a new aliquot of acid-ethanol solution, and insulin was reextracted overnight. Following centrifugation, the supernatant was collected and pooled with the first extracted sample. Finally, the insulin concentration was measured using a mouse-specific ELISA kit (Ultrasensitive Mouse Insulin ELISA, Mercodia, Uppsala, Sweden).

### 2.12. Statistical Analysis

Data are expressed as mean ± standard error. Statistical differences between means were analyzed using a paired or unpaired Student's *t*-test or one-way ANOVA as appropriate. The cumulative cure rate in groups A and B was expressed using Kaplan-Meier plots. The log-rank test was used to analyze differences in cure rate between groups A and B. A *p* value < 0.05 was considered to be statistically significant.

## 3. Results

### 3.1. Achievement of Full and Low-Grade Donor Chimerism of Hematopoietic Cells

A state of high-grade (>60%) multilineage donor chimerism of the hematopoietic cells of C57BL/6 (H2^b^) origin was rapidly established in the conditioned diabetic BALB/c (H2^d^) mice in both group A (renal site) and group B (subcutaneous site) at 2 weeks post-BMC transplantation (Table 1). At 8 weeks after BMC transplantation, stable full donor chimerism (>95%) was achieved in all of the mice in groups A and B. There were no differences in the speed or extent of donor chimerism establishment in those mice that received subcutaneous implantation of islets (group B) compared to those that received renal implantation of islets (group A). Mice in group C did not receive allogeneic BMCs, and no hematopoietic cells of C57BL/6 (H2^b^) origin were detected. Compared to the mice in group B, those in group D had low-grade (<5%) donor chimerism at 2 weeks, and low-grade mixed chimerism was maintained for at least 8 weeks postislet transplantation.

### 3.2. Successful Achievement of Full Multilineage Donor Chimerism Was Associated with a Preceding Higher Expansion of CD11b^+^ and Gr-1^+^ Myeloid Progenitor Cells and Effector Memory CD4 and CD8 T Cells

The time frame of myeloid cell development during the establishment of chimerism revealed that more than 80% of the peripheral myeloid cells were CD11b^+^ and Gr1^+^ cells at 2 weeks after BMC transplantation in the mice in groups A and B which had established high-grade donor chimerism (Table 2). The percentages of CD11b^+^ and Gr1^+^ cells still represented 50% of the peripheral myeloid cells at 8 weeks post-BMC transplantation in both groups A and B, which were significantly higher than the percentages of CD11b^+^ and Gr1^+^ cells in healthy C57BL/6 mice (CD11b: 17.3 ± 4.0% and Gr1: 14.3 ± 1.8%). Mice in group D that had received both BMCs and WJ-MSCs had significantly fewer CD11b^+^ and Gr-1^+^ cells and achieved a low-grade donor chimerism at 2 weeks posttransplantation and thereafter (Table 2). At 2 and 8 weeks, mice in group B had significantly fewer CD11c^+^ cells than that of mice in group A (Table 2). The characteristics of T cell subsets were analyzed, and a significant elevation of both effector memory CD4 and CD8 T cells was noted in both groups A and B compared to group D at 2 weeks posttransplantation (Table 3).

### 3.3. Full Multilineage Donor Chimerism Was Associated with Successful Engraftment of Allogeneic Islets and Restoration of Normoglycemia

**Figure 2:**
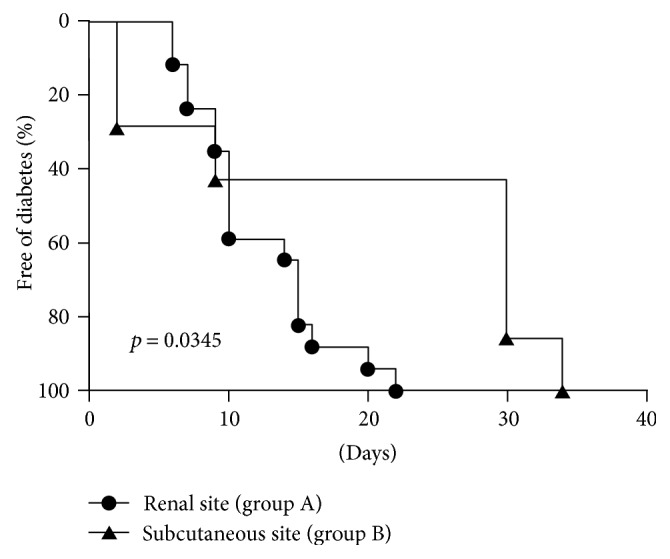
Kaplan-Meier plot of cumulative cure rate of diabetes. Diabetic mice in both groups A and B received conditioning and BMC transplantation. The mice in group A received 300 allogeneic islets under the left kidney subcapsular space, and the mice in group B received 900 allogeneic islets under the subcutaneous space of the back. After islet transplantation, nonfasting whole blood glucose levels of the recipients were determined twice weekly, and the cure of diabetes was defined as a whole blood glucose level below 200 mg/dL for two consecutive tests and thereafter. The difference in cumulative cure rate of diabetes between two the groups was analyzed with the log-rank test, and the *p* value of the comparison was <0.05.

**Figure 3:**
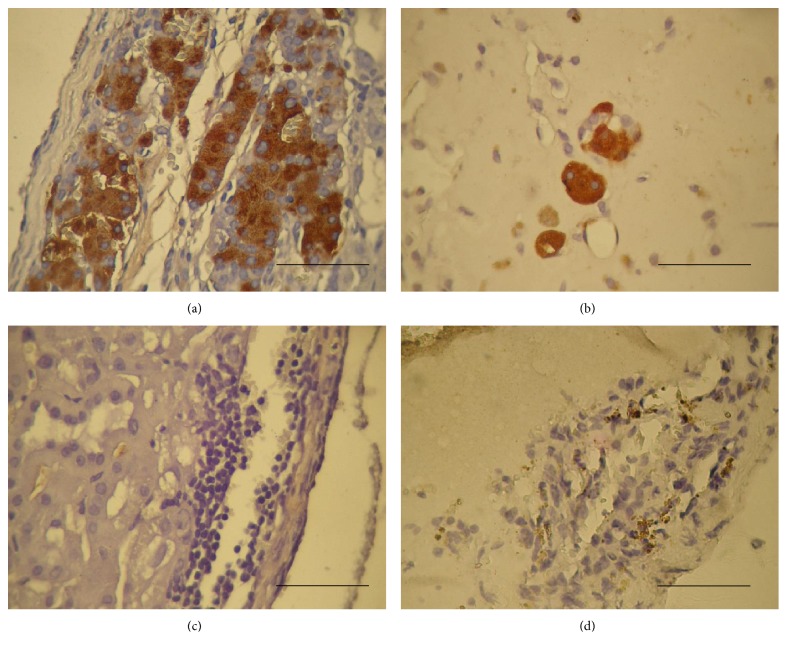
Immunohistochemical examination of the retrieved islet grafts. The graft-bearing kidneys and the subcutaneous grafts from the mice in groups A, B, C, and D were retrieved at 20 to 30 weeks posttransplantation. The immunohistochemical examinations were performed using anti-insulin antibodies. The secondary antibody was prepared from rabbits and conjugated with horseradish peroxidase, and diaminobenzidine was used as the chromogenic substance. The dark-brownish precipitates indicate insulin-containing cells. (a), (b), (c), and (d) show the graft histology of mice in groups A, B, C, and D, respectively. Photos are 40 × 10 magnification. Scale bars indicate 0.1 mm.

The duration of temporary hyperglycemia in the mice in group A that received 300 islets under the renal capsule was significantly shorter than that in the mice in group B that received 900 islets subcutaneously (10.8 ± 1.2, *n* = 16, versus 18.9 ± 5.3, *n* = 8, *p* < 0.05). Kaplan-Meier plots and log-rank analysis revealed that the cumulative cure rate of diabetes in group A was achieved significantly faster than in group B (*p* < 0.05) (Figure 2()). Groups C and D maintained hyperglycemia and were not cured of diabetes during the entire observation period. The protective effect of full multilineage donor chimerism on donor islet cells was further supported by the existence of viable islet beta cells in the grafts of mice in groups A and B that were retrieved at 20 to 30 weeks postislet transplantation (Figures 3() and 3(b)). But there were no viable islet beta cells in the graft of mice in groups C and D (Figures 3() and 3(d)). The insulin content of the pancreatic remnants was measured at sacrifice and was found to be 483 ± 90 ng (*n* = 8), 507 ± 102 ng (*n* = 4), 520 ± 98 ng (*n* = 4), and 372 ± 120 ng (*n* = 4) for the mice in groups A, B, C, and D, respectively. The pancreatic insulin content for the healthy male BALB/c mice at 16 weeks of age was 3250 ± 310 ng (*n* = 6). Our results demonstrated that the restoration of normoglycemia was resulted from successful allogeneic islet engraftment in full donor chimerism groups rather than pancreas regeneration.

### 3.4. The Impact of Donor Chimerism on the Profile of Blood Glucose and Body Weight Changes

**Figure 4:**
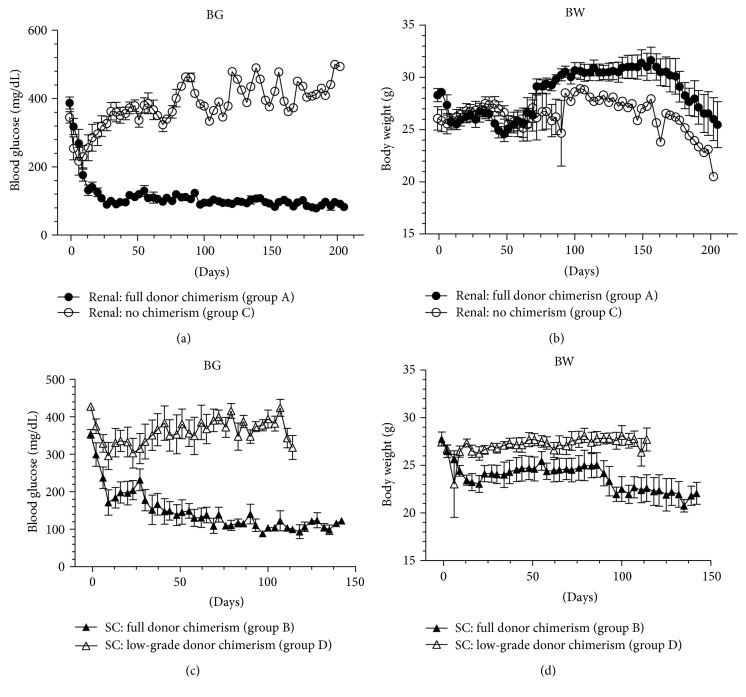
The effect of donor chimerism on blood glucose and body weight changes in the mice. All mice in groups A and C received 300 allogeneic islets under the left kidney subcapsular space. The mice in group A received conditioning and BMC transplantation, whereas the mice in group C received conditioning but no BMC infusion. The nonfasting whole blood glucose level (a) and body weight (b) of the mice in groups A and C were determined twice weekly. All mice in groups B and D received 900 allogeneic islets under the subcutaneous space of the back. The mice in group B received conditioning and BMC transplantation, whereas the mice in group D received conditioning and transplantation of both BMCs and MSCs. The nonfasting whole blood glucose level (c) and body weight (d) of the mice in groups B and D were determined twice weekly.

Full donor chimerism of hematopoietic cells in group A enhanced engraftment of renal subcapsular donor islets and restored normoglycemia at 2 weeks posttransplantation (basal: 387 ± 18 mg/dL versus 2 weeks: 131 ± 15 mg/dL, *p* < 0.005) (Figure 4(a)). Mice in group C that had no donor chimerism had an initial decrease in blood glucose; however, their blood glucose levels increased rapidly and were consistently higher than 360 mg/dL thereafter. The body weight gain of the mice in group A was significantly higher than that of the mice in group C (Figure 4(b)). Furthermore, full donor chimerism also protected the subcutaneous allogeneic islets of the mice in group B (Figure 4(c)). Normoglycemia was restored in the mice in group B at 2 weeks posttransplantation (basal: 354 ± 12 mg/dL versus 2 weeks: 184 ± 28 mg/dL, *p* < 0.05). However, low-grade donor chimerism did not protect the subcutaneously implanted allogeneic islets from being rejected, as shown in the mice in group D that maintained hyperglycemia after islet transplantation (Figure 4(c)). Interestingly, the body weight of the mice in group B that had full donor chimerism and normoglycemia was significantly lower than that of the mice in group D that had low-grade donor chimerism and hyperglycemia (Figure 4(d)).

## 4. Discussion

### 4.1. Full but Not Low-Grade Donor Chimerism Restored Normoglycemia in the Diabetic Mice That Received Subcutaneous Transplantation of Allogeneic Islets

Bethge et al. previously analyzed the outcomes in patients that received donor lymphocyte infusion (DLI) for low or falling chimerism after nonmyeloablative conditioning for allogeneic hematopoietic cell transplantation. They found that patients who remained low donor CD3 chimerism (<5%) eventually rejected their grafts, suggesting that low donor CD3 chimerism appeared to predict rejection [11]. However, it has been reported that low-grade donor chimerism can protect kidney grafts without the administration of immunosuppressants in allogeneic kidney transplantation [12]. Our study revealed that only full but not low-grade donor chimerism restored normoglycemia in diabetic mice that received subcutaneously implanted allogeneic islets. It is not clear why low-grade donor chimerism could not restore normoglycemia in the treated diabetic mice. However, Biarnes et al. and Gala-Lopez et al. reported that early functional engraftment failure and primary nonfunctioning destroyed more than 60% of the implanted islet cells and that inadequate protection of islet cells by low-grade donor chimerism in the first 2 weeks of transplantation would further reduce viable islet cells thereby resulting in graft failure and hyperglycemia [13, 14]. Since the first 2 weeks of transplantation is the golden period of time for islet engraftment, rapidly achieving full donor chimerism may provide allogeneic islet cells with full protection and then restore normoglycemia [15].

### 4.2. The Site of Islet Transplantation Did Not Affect the Achievement of Full Donor Chimerism Which Was Preceded by a High Transient Expansion of CD11b^+^ and Gr-1^+^ Myeloid Progenitor Cells Post-BMC Transplantation

In this study, full multilineage donor chimerism was achieved rapidly after two infusions of BMCs into the conditioned diabetic BALB/c mice regardless whether that islets were implanted into the subcutaneous or renal subcapsular space. The achievement of full donor chimerism was associated with a higher transient expansion of CD11b^+^ and Gr-1^+^ myeloid progenitor cells and effector memory CD4 and CD8 T cells in both the subcutaneous and renal subcapsular islet transplantation groups. Our results suggest that the site of islet transplantation did not affect the establishment of donor chimerism using nonmyeloablative conditioning and BMC transplantation. Previous study has shown that induction of bone marrow chimerism in irradiated mice is associated with a transient expansion of CD11b^+^ Gr-1^+^ cells, defined as myeloid-derived suppressor cells (MDSC) in mice, with in vitro T cell suppressive activity [16]. In our study, such regulatory myeloid cells were arisen and may regulate posttransplant T cell alloreactivity in full multilineage donor chimerism groups. Furthermore, the rapid increase of TEMs of CD4 and CD8 is possible to replace the host alloreactive memory T cells and to prevent immune rejection of the donor islet grafts, supporting the hypothesis that preexisting memory T cells in the alloreactive repertoire is a major obstacle to the induction and maintenance of tolerance and that the only agents that can eliminate alloreactive host T cells are alloreactive donor T cells [17]. When the MSCs and BMCs were coadministered, the immunomodulatory effect of MSCs increased the percentages of CD11c^+^ cells (dendritic cells) and Foxp3^+^CD4 cells (Treg), significantly suppressed transient expansion of CD11b^+^ and Gr-1^+^ myeloid progenitor cells, and established stable low-grade multilineage donor chimerism in the conditioned mice. Our data suggest that a high transient expansion of CD11b^+^ and Gr-1^+^ myeloid progenitor cells precedes the achievement of full donor chimerism.

### 4.3. The Influence of Graft-versus-Host Disease on the Treated Diabetic Mice

Graft-versus-host disease (GvHD) is a pathologic attack by donor T cells on normal host tissues including the skin, liver, and gastrointestinal tract. Mohty et al. found that those who have full donor T cell chimerism at day 30 had a higher incidence of grades 2–4 acute GvHD compared with mixed T cell chimerism cases [18]. Since the body weight of the successfully transplanted diabetic mice increased gradually (by 3% and 6% of their initial weight at 3 and 6 weeks postislet transplantation, respectively), the gradual loss of body weight after BMC transplantation in groups A and B may suggest ongoing graft-versus-host disease despite no obvious symptoms such as diarrhea, skin lesions, or hair loss. The body weight of the mice that received renal subcapsular islet transplantation decreased initially and then increased from 6 weeks post-BMC and islet transplantation. Compared to the mice that received renal subcapsular islet transplantation, the mice that received subcutaneous islets did not obviously gain body weight after BMC and islet transplantation. Although the CD11c^+^ cells have been postulated to be required to initiate and maintain GvHD [19], there is no significant correlation between the chimerism and percentage of CD11c^+^ cells and GvHD [20]. In this study, compared with the renal group, the cured diabetic mice receiving subcutaneous islet transplantation had a lower percentage of CD11c^+^ cells but its clinical significance is not clear. Whether the differences in body weight change between the mice that received subcutaneous islets and those that received renal subcapsular islets were related to the degree of graft-versus-host disease is unclear.

In conclusion, full donor chimerism protected both renal subcapsular and subcutaneous allogeneic islets. Using the subcutaneous site for islet transplantation, many more islets and rapidly achieving full donor chimerism were required to restore normoglycemia. Recipient mortality due to graft-versus-host disease and/or loss of body weight offsets the prolonged survival of allogeneic islets from donor chimerism without immunosuppressive treatment.

## Figures and Tables

**Table 1:** Donor chimerism achieved in the peripheral blood of mice that received conditioning and bone marrow cell and/or mesenchymal stem cell transplantation.

Donor chimerism (%)	Group A Renal site (*n* = 16)		Group B Subcutaneous site (*n* = 8)		Group D Subcutaneous site (*n* = 6)	
Time	2 weeks	8 weeks	2 weeks	8 weeks	2 weeks	8 weeks
CD4	61.6 ± 8.9	98.8 ± 0.5	69.4 ± 12.9	98.7 ± 1.2	0.3 ± 0.1	1.6 ± 0.5
CD8	94.0 ± 0.9	97.2 ± 0.4	91.2 ± 3.4	98.3 ± 0.9	4.8 ± 3.0	4.1 ± 1.7
CD45	94.3 ± 2.1	99.0 ± 0.4	94.8 ± 5.2	99.7 ± 0.1	3.3 ± 3.1	0.1 ± 0.0
CD11b	94.2 ± 0.8	96.6 ± 2.6	94.4 ± 4.4	98.8 ± 0.2	3.8 ± 3.6	0.8 ± 0.2
CD11c	56.6 ± 8.3	81.5 ± 1.5	62.3 ± 4.4	74.1 ± 3.0	2.1 ± 1.1	1.9 ± 0.7
CD19	90.5 ± 2.6	98.6 ± 0.1	86.8 ± 9.1	98.2 ± 0.7	2.4 ± 2.0	2.1 ± 0.7
Gr1	90.5 ± 1.4	96.2 ± 1.2	92.6 ± 5.0	97.8 ± 0.3	2.3 ± 2.0	1.8 ± 0.4
Foxp3	30.8 ± 9.3	58.1 ± 1.6	38.1 ± 1.4	50.8 ± 1.8	9.1 ± 3.4	9.4 ± 0.4

Chimerism in the BALB/c mice was detected by staining for H-2D^b^-expressing (C57BL/6 donor) cells that were distinguishable from BALB/c cells expressing H-2D^d^ at 2 and 8 weeks posttransplantation. Other antibodies used for chimerism analysis included CD45, CD4, CD8, CD11b, CD11c, CD19, Ly6G/6C (Gr1), and Foxp3. All comparisons between group A and group B did not differ. All comparisons between group B and group D were statistically significant and had a *p* value of less than 0.001.

**Table 2:** Population of different cells developed in the peripheral blood of mice that received conditioning and bone marrow cell and/or mesenchymal stem cell transplantation.

Cell population (%)	Group A Renal site (*n* = 16)		Group B Subcutaneous site (*n* = 8)		Group D Subcutaneous site (*n* = 6)	
Time	2 weeks	8 weeks	2 weeks	8 weeks	2 weeks	8 weeks
CD4	2.8 ± 1.0	9.7 ± 1.9	2.3 ± 1.3^i^	7.1 ± 2.8^o^	15.2 ± 2.8^i^	17.9 ± 1.6^o^
CD8	8.5 ± 1.9	3.6 ± 0.5	5.9 ± 1.0	4.5 ± 0.7	6.8 ± 1.8	6.3 ± 0.8
CD45	93.1 ± 1.5	92.8 ± 1.8	91.9 ± 1.0	89.8 ± 1.9	88.9 ± 1.3	98.2 ± 0.3
CD11b	83.4 ± 1.5	48.3 ± 6.6	85.7 ± 2.8^j^	53.3 ± 14.7	57.4 ± 2.7^j^	44.6 ± 4.9
CD11c	9.0 ± 2.2^a,b^	6.2 ± 1.1^e,f^	3.9 ± 0.6^a,k^	1.1 ± 0.2^e,p^	11.8 ± 4.3^b,k^	9.6 ± 2.5^f,p^
CD19	1.1 ± 0.2^c^	15.7 ± 2.3^g^	5.8 ± 1.7^l^	17.7 ± 5.5	15.7 ± 4.7^c,l^	19.9 ± 4.9^g^
Gr1	81.2 ± 1.5	45.1 ± 6.8	81.1 ± 3.3^m^	49.9 ± 15.4	61.6 ± 5.1^m^	54.0 ± 5.0
Foxp3	4.9 ± 0.8^d^	6.5 ± .0.5^h^	5.3 ± 1.2^n^	5.9 ± 1.2^q^	10.4 ± 1.9^d,n^	10.7 ± 0.6^h,q^

The mean frequency of different cells in the venous blood was measured by flow cytometry at 2 and 8 weeks posttransplantation. ^a,b,f,g,l,n^*p* < 0.05, ^d,e,h,k,o,p^*p* < 0.01, ^i,m,q^*p* < 0.005, and ^c,j^*p* < 0.001 comparing the two means using independent *t*-tests.

**Table 3:** T cell subsets were identified by flow cytometry analysis.

T subset (%)	Group A Renal site (*n* = 16)		Group B Subcutaneous site (*n* = 8)		Group D Subcutaneous site (*n* = 6)	
Time	2 weeks	8 weeks	2 weeks	8 weeks	2 weeks	8 weeks
CD4-TN	31.1 ± 5.4	66.5 ± 9.2	38.4 ± 11.3	59.6 ± 15.9	60.8 ± 7.9	31.2 ± 9.5
CD4-TEM	37.7 ± 5.5	14.6 ± 8.9	35.8 ± 8.6^a^	24.6 ± 13.8	12.0 ± 3.8^a^	15.3 ± 1.9
CD4-TCM	5.5 ± 1.0	9.9 ± 1.5	5.6 ± 1.0	9.1 ± 1.6	8.7 ± 1.0	9.2 ± 2.5
CD8-TN	1.4 ± 0.3	47.4 ± 9.5	3.4 ± 1.5^b^	34.4 ± 15.9	15.7 ± 2.8^b^	12.4 ± 4.3
CD8-TEM	90.7 ± 0.9	29.6 ± 9.4	86.0 ± 1.9^c^	49.9 ± 17.2	41.9 ± 6.1^c^	31.8 ± 3.8
CD8-TCM	4.3 ± 0.4	16.0 ± 1.5	7.9 ± 1.2^d^	13.9 ± 2.4	21.7 ± 3.5^d^	22.2 ± 3.3

Naive T cells (TN), central memory T cells (TCM), and effector memory T cells (TEM) were defined as CD44^lo^CD62L^+^, CD44^hi^CD62L^+^, and CD44^hi^CD62L^−^, respectively. ^a^*p* < 0.05, ^b^*p* < 0.005, ^c^*p* < 0.001, and ^d^*p* < 0.01 comparing the indicated two means using independent *t*-test.
